# Scrutinizing the drug resistance mechanism of multi- and extensively-drug resistant *Mycobacterium tuberculosis:* mutations versus efflux pumps

**DOI:** 10.1186/s13756-019-0516-4

**Published:** 2019-05-02

**Authors:** Hasan Ghajavand, Mansour Kargarpour Kamakoli, Sharareh Khanipour, Shahin Pourazar Dizaji, Morteza Masoumi, Fatemeh Rahimi Jamnani, Abolfazl Fateh, Mehdi Yaseri, Seyed Davar Siadat, Farzam Vaziri

**Affiliations:** 1 Department of Mycobacteriology and Pulmonary Research, Pasteur Institute of Iran, Tehran, Iran; 2 Microbiology Research Center (MRC), Pasteur Institute of Iran, No. 358, 12th Farvardin Ave, Jomhoori St, Tehran, 1316943551 Iran; 3 Department of Epidemiology and Biostatistics, Tehran University of Medical Sciences, Tehran, Iran

**Keywords:** Tuberculosis, VP, Efflux pumps, MDR, Pre-XDR, XDR

## Abstract

**Background:**

In order to shorten the course of treatment and its effectiveness, it is essential to gain an in-depth insight into the drug resistance mechanisms of *Mycobacterium tuberculosis* (*M. tuberculosis*).

**Methods:**

In this study, we evaluated the contribution of 26 drug efflux pumps plus target gene mutations to the drug resistance levels in multi-drug resistant (MDR)/pre-extensively drug-resistant (pre-XDR)/extensively drug-resistant (XDR) and mono-drug resistant clinical isolates of *M. tuberculosis*. The panels of 25 *M. tuberculosis* clinical strains were characterized for drug resistance-associated mutations with whole-genome sequencing and antibiotic profiles in the presence and absence of efflux inhibitor verapamil (VP).

**Results:**

Different MICs were observed for the same target gene mutations. Out of the 16 MDR/pre-XDR/XDR isolates, 6 (37.5%) and 3 (18.8%) isolates demonstrated a significant decrease in rifampicin (RIF) MIC and isoniazid (INH) MIC due to the VP exposure (64 μg/mL), respectively. Susceptibility to RIF was fully restored in two isolates after VP exposure. Moreover, the efflux pump genes of *Rv2938, Rv2936, Rv1145, Rv1146, Rv933, Rv1250, Rv876*, *Rv2333, Rv2459, Rv849,* and *Rv1819* were overexpressed in the presence of anti-TB drugs, showing the contribution of these efflux pumps to the overall resistance phenotype.

**Conclusions:**

Our results clearly showed that efflux systems, besides spontaneous mutations, play a role in the development of INH/RIF resistance. In addition, although VP was effective in reducing the expression of some efflux pumps, it was not very successful at the phenotypic level.

**Electronic supplementary material:**

The online version of this article (10.1186/s13756-019-0516-4) contains supplementary material, which is available to authorized users.

## Background

In recent years, tuberculosis (TB) has threatened communities all over the world and it is still one of the major public health concerns in many countries [1]. According to the latest report of the World Health Organization, the global incidence rate of TB is approximately 10 million cases, of which 5.8 million (58%) are men, 3.2 million (32%) are women, and 1.0 million (10%) are children [2]. Given the limited number of available anti-TB drugs, the emergence of multidrug-resistant TB (MDR-TB) and extensively drug-resistant TB (XDR-TB) has increased the complexity of designing appropriate treatment regimens. MDR-TB is caused by *Mycobacterium tuberculosis* (*M. tuberculosis*) that is resistant at least to isoniazid (INH) and rifampicin (RIF) while XDR-TB is caused by mycobacteria resistant to RIF and INH, along with fluoroquinolone and one of the three injectable drugs, namely capreomycin, kanamycin, and amikacin [3]. Resistance to anti-TB drugs is caused mainly by mutations in drug target genes [4], the impermeability of *M. tuberculosis* cell wall, and the activity of efflux pumps [5, 6]. The presence of mutations in the target genes of antibiotics is considered the most important resistance mechanism in this bacterium [7].

Other mechanisms of resistance, such as efflux pumps, act synergistically with the permeability barrier to reduce the passage of antimicrobials across the bacterial outer membrane [8]. Previous studies have demonstrated that the resistance of *M. tuberculosis* is associated with constitutive or inducible expression of efflux systems [9, 10]. Efflux pumps utilize the transmembrane electrochemical gradient of protons or sodium ions to extrude drugs from the cell, thereby neutralizing drug activity [11]. Efflux pumps are classified into six categories, including major facilitator superfamily (MFS), ATP-binding cassette (ABC), small multidrug resistance (SMR), resistance–nodulation–division (RND), multidrug and toxic compound extrusion (MATE), and proteobacterial antimicrobial compound efflux (PACE) [12, 13]. MFS, ABC, RND, and SMR efflux pumps have been found in *M. tuberculosis* [14]. Efflux pumps usually confer low levels of drug resistance but play a significant role in evolving to high levels of resistance in *M. tuberculosis* [15].

Recently, efflux pump inhibitors (EPIs) have been demonstrated as a putative new drug compound, since these types of molecules bind to bacterial efflux pumps to inhibit their efflux function [16]. EPIs binding to *M. tuberculosis* efflux pumps were shown to inhibit the efflux of anti-TB drugs, enhance *M. tuberculosis* killing, reverse *M. tuberculosis* drug resistance, and produce synergistic effects with first-line anti-TB drugs [17, 18]. Of the EPIs evaluated, verapamil (VP) has shown the most potent efflux inhibition. Studies with INH- or RIF-resistant clinical isolates demonstrated that the combined use of VP with INH or RIF reduced the minimum inhibitory concentration (MIC) of both drugs and reversed *M. tuberculosis* drug resistance against both drugs [19, 20].

In the current study, we (i) determined the MICs of anti-TB drugs, (ii) investigated the effect of VP on the MICs, and (iii) evaluated the expression of 26 genes encoding putative drug efflux pumps in selected MDR/pre-XDR/XDR and mono-resistant *M. tuberculosis* isolates.

## Methods

### Bacterial strains and mutation analysis

In this retrospective study 25 clinical isolates were used, 16 of which were MDR/pre-XDR/XDR and 9 isolates were mono-drug resistant (3 mono-RIF, 3 mono-INH, and 3 mono-EMB resistant isolates). H37Rv strain and nine pan-susceptible clinical strains were also studied for comparison purposes. All of these isolates were collected, from January 2014 to January 2018, at the Department of Mycobacteriology and Pulmonary Research, Pasteur Institute of Iran. Whole genome sequencing data of all the isolates were available from our previous study [21]. The Ethics Committee of Pasteur Institute of Iran performed the ethical reviews, and written informed consents were obtained from the participants.

### Antimicrobial, EPI, and MIC agents

Middlebrook 7H9 broth and albumin-dextrose-catalase (ADC) supplement were purchased from Difco (Detroit, MI, USA). INH, RIF, ethambutol (EMB), streptomycin (STR), ofloxacin (OFX), kanamycin (KAN), capreomycin (CAP), and VP were obtained from Sigma-Aldrich (St. Louis, MO, USA). All the solutions were prepared on the day of the experiment. Alamar blue was obtained from AbD Serotec (Oxford, UK).

### Conventional drug susceptibility testing

Clinical isolates were re-confirmed for susceptibility to four first-line anti-TB drugs (i.e., INH, RIF, STR, and EMB) and three second-line anti-TB drugs (i.e., KAN, OFX, and CAP) using a proportion method with Lowenstein–Jensen medium as described by the World Health Organization [19]. The drug concentrations in the medium were as follows: 0.2 μg/mL INH, 40 μg/mL RIF, 4 μg/mL STR, 2 μg/mL EMB, 30 μg/mL KAN, 2 μg/mL OFX, and 40 μg/mL CAP [22].

### Determination of MICs and VP effectiveness

A microplate Alamar blue assay was performed as previously described to determine the MICs of all the 25 clinical isolates [23]. The effects of VP on the MICs of INH and RIF (for MDR/pre-XDR/XDR isolates) and INH, RIF, and EMB (for respective mono-resistant isolates) were also studied by incorporating the inhibitor at sub-inhibitory concentrations in *M. tuberculosis* cultures in the assay. Two-fold serial dilutions of RIF (concentration range of 0.001–128 μg/mL), INH (0.001–256 μg/mL), and EMB (0.1–50 μg/mL) were made directly in the wells in the absence or presence of 64 μg/mL of VP. The concentration of VP (16–128 μg/mL) was determined after studying the effect of concentration-dependent titration using this inhibitor on a certain MDR isolate. In addition, in a previous study, the 64 μg/mL concentration was proven as the ideal concentration [24].

MIC was defined as the lowest drug concentration preventing a change in color. Isolates with MICs of INH < 0.25 μg/ml, RIF < 1 μg/ml, and EMB ≤ 2.5 μg/ml were defined as being susceptible to INH, RIF, and EMB, respectively [23]. All the tests for each strain were carried out at least in duplicate to calculate the mean MIC for each strain.

### Expression profile of drug efflux pumps

To extract the total bacterial RNA, *M. tuberculosis* clinical isolates were cultured in 10 mL of Middlebrook 7H9 broth (BD) with ADC supplement for the 16 MDR/pre-XDR/XDR and 9 mono-drug resistant isolates. RIF, INH, EMB, RIF + VP, INH + VP, and EMB + VP were added to these cultures individually at sub-inhibitory concentrations (half of the MIC), incubated at 37 °C for 25 days, and collected for RNA extraction. The total bacterial RNA was isolated using PREP-NA DNA/RNA extraction kit according to the manufacturer’s instructions. The quality and integrity of total RNA were assessed using a nanophotometer.

After treatment with DNase I (Invitrogen), RNA (1 μg) was submitted to cDNA synthesis according to the manufacturer’s recommendations (PrimeScript™ 1st strand cDNA Synthesis Kit, TAKARA). Quantitative reverse transcription PCR was performed in a 20-μl system containing 10 μl of 2 × mixture supplied with SYBR Green, 100 ng of complementary DNA template, and 5 pmol of each primer set. The primer sets and sequences of oligonucleotides are described elsewhere [24]. To assure specific amplification, the melting curves of each reaction were assessed and each sample was performed in duplicate. *polA* and *secA* were used as the housekeeping genes for normalization. The quantification of target gene expression in induced strains relative to non-induced strains was performed by the 2^−ΔΔCt^ method using the GenEx 6 software [25]. An expression equal to 1 indicated identical expression levels, an expression ⩾ 4 indicated up-regulation, and an expression ≤4 indicated down-regulation [24].

### Statistical analysis

The Wilcoxon signed-rank test was used to evaluate the expression changes in the same isolate due to exposure to VP (in addition to the conventional drug). We used the linear mixed model (LMM) to assess the difference in the expression rates of various types of genes within the same isolate. All the statistical analyses were performed in IBM SPSS statistics for Windows version 25.0 software (IBM Corp. Released 2017, Armonk, NY). A *P*-value of less than 0.05 was considered statistically significant.

## Results

### MICs and the effect of VP

Among the 16 MDR/pre-XDR/XDR-TB clinical isolates, PII-30 and PII-33 were resistant to all the antibiotics tested, 10 isolates were resistant to all the first-line drugs (i.e., STR, INH, RIF, and EMB), and the remaining isolates were resistant to at least two of the first-line drugs. The INH MICs of the 16 MDR/pre-XDR/XDR isolates varied from 2 to 64 μg/mL, whereas the RIF MICs varied from 4 to 128 μg/mL (Table 1).

**Table 1:**
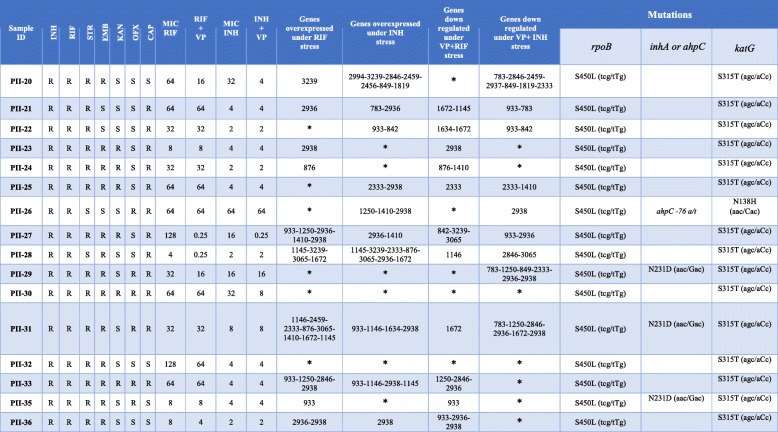
Drug susceptibility profile, MIC results, VP treatment, efflux pump expression and mutations in 16 MDR/pre-XDR and XDR isolates

*: not changed

*INH* Isoniazid, *RIF* Rifampicin, *EMB* Ethambutol, *CAP* Capreomycin, *KAN* Kanamycin, *STR* Streptomycin, *OFX* Ofloxacin, *VP* Verapamil, *S* Susceptible, *R* Resistant, *MIC* Minimum inhibitory concentration

Out of the 16 MDR/pre-XDR/XDR isolates, 6 (37.5%) and 3 (18.8%) isolates demonstrated significant reductions (*P* < 0.05) in RIF MICs and INH MICs after VP exposure, respectively. Susceptibility to RIF was fully restored (MIC = 0.25 μg/mL) in two isolates (i.e., PII-27 and PII-28) after VP exposure.

Of the nine mono-drug resistant clinical isolates, three were resistant to RIF, three were resistant to INH, and three were resistant to EMB. The highest MIC (128 μg/mL) was related to the PII-4 strain (mono-RIF resistant isolate). Only in PII-15 (mono-EMB resistant isolate) was observed a two-fold decrease in MIC in the presence of VP (Table 2).

**Table 2:**
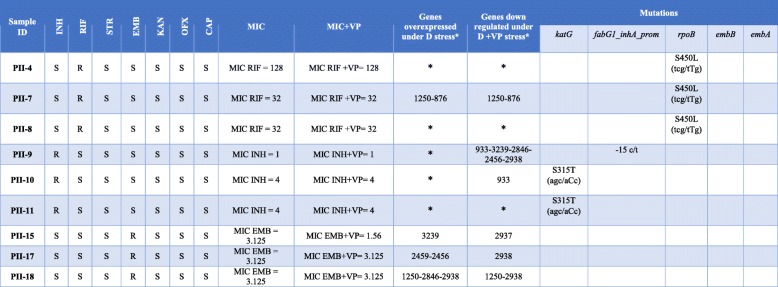
Drug susceptibility profile, MIC results, VP treatment, efflux pump expression and mutations in 9 mono drug resistant isolates

*: not changed

*D Drug, *INH* Isoniazid, *RIF* Rifampicin, *EMB* Ethambutol, *CAP* Capreomycin, *KAN* Kanamycin, *STR* Streptomycin, *OFX* Ofloxacin, *VP* Verapamil, *S* Susceptible, *R* Resistant, *MIC* Minimum inhibitory concentration

### Gene mutation analysis

The whole genome sequencing revealed that all the MDR/pre-XDR/XDR-TB isolates had the *katG* S315 T mutation, except PII-26 that was identified to have a combination of *katG* N138H and *ahpC* t-76a mutations and the highest MIC (64 μg/mL) compared to other MDR/pre-XDR/XDR strains. The RIF resistance in all the MDR/pre-XDR/XDR strains was attributed to mutations in the *rpoB* hot-spot region (S450 L mutation) (Table 1). Among the nine mono-resistant clinical isolates, all the mono-RIF resistant isolates had the S450 L mutation in *rpoB*. Regarding the three mono-INH resistant isolates, only harbored two isolates the *katG* S315 T mutation, and PII-9 did not have any related mutations. No mutations at *embA* or *embB* were detected in the mono-EMB resistant clinical isolates (Table 2).

### Expression profile of drug efflux pumps

According to Table 1, 10 MDR/pre-XDR/XDR isolates had at least one gene overexpressed under RIF and INH stress (⩾four-fold induction). Among the 16 MDR/pre-XDR/XDR isolates, there were two isolates (PII-31 and PII-20) and one isolate (PII-28) with the overexpression of eight and seven efflux pump genes under RIF and INH stress, respectively. In addition, 11 and 9 isolates under RIF + VP and INH + VP stress showed a four-fold down-regulation in at least one of these 26 studied genes, respectively.

Among the nine mono-drug resistant clinical isolates, PII-4, PII-8, PII-9, PII-10, and PII-11 showed no induction in any of the 26 efflux pump genes under RIF and INH stress. Of the nine mono-drug resistant isolates, four isolates overexpressed one or two of the following genes: *Rv1250, Rv876, Rv3239, Rv2459, Rv2456, Rv2846*, and *Rv2938*. Moreover, among the 26 tested efflux pump genes, *Rv2938* was down-regulated more than four folds under RIF + VP, INH + VP, and EMB + VP stress in three isolates (Table 2).

Additional data regarding drug susceptibility, MIC results, VP treatments, efflux pump expression, and mutations are provided in Table 1 (for MDR/pre-XDR/XDR) and Table 2 (for mono-resistant isolates). The differential expression of efflux pump genes is provided in Additional file 1: Tables S1 and S2.

## Discussion

In order to shorten the course of treatment and its effectiveness, it is essential to gain an in-depth insight into the drug resistance mechanisms of *M. tuberculosis* [26]. Although drug resistance is acquired mainly due to mutational modifications of the drug target, it has become clear that multi-drug efflux systems may play roles in the drug resistance of *M. tuberculosis,* as well [27]*.*

In the current study conducted at the phenotypic level, an efflux pump inhibitor, VP, exerted a significant effect on the reduction of RIF MIC and INH MIC in 37.5 and 18.8% of the MDR/pre-XDR/XDR isolates, respectively. Two isolates (PII-27 and PII-28) merely showed susceptibility to RIF after the VP exposure. Among the mono-resistant isolates, the MIC reduction in the presence of VP was observed in only one mono-EMB resistant isolate (i.e., PII-15). Collectively, the current study showed that VP partially restored the potency of RIF, INH, and EMB against drug-resistant *M. tuberculosis* isolates but not as successful as previous studies [16, 24, 28]. Recently, it was shown that VP does not affect intracellular anti-TB drug uptake and accumulation in *M. tuberculosis* through the direct inhibition of efflux pumps; instead, it targets membrane energetics in the bacterium [29]. However, this issue needs further investigations.

We investigated mutations in target genes, the expression levels of 26 genes encoding putative drug efflux transporters under drug stress, and efflux inhibition with VP at the mRNA level. Most of the MDR/pre-XDR/XDR isolates in our study had the *katG* S315 T and *rpoB* S450 L mutations. It seems that these mutations play a pivotal role in INH and RIF resistance, respectively. As demonstrated in Table 1, MDR/pre-XDR/XDR isolates were similarly (10 vs. 10) induced by RIF and INH to overexpress efflux pump genes, but more genes were induced by INH than by RIF (34 vs. 28). Li et al. reported that the expression levels of genes in response to INH were significantly higher in MDR than in RIF-resistant isolates. Additionally, more genes were expressed in response to INH compared to RIF [30], suggesting that more efflux pumps may respond to INH than to RIF in MDR/pre-XDR/XDR *M. tuberculosis*. Our study showed that some of these genes fail to be up-regulated in any of the MDR/pre-XDR/XDR isolates after RIF/INH treatment. In addition, Li et al. reported that *Rv1258 (tap)* and *Rv2265* by INH and *Rv783 (emrB), Rv1258, Rv2994, Rv2456, Rv2265,* and *Rv*849 by RIF were not overexpressed in any of the MDR isolates, which was consistent with the results of our study [30].

ABC transporters constitute a large superfamily of proteins, which are able to import or export a wide range of substances, including amino acids, ions, sugars, lipids, and drugs. In addition, they are involved in the determination of intrinsic levels of resistance in *M. tuberculosis* [31, 32]. The *drr* (doxorubicin resistance) operon was first identified in *Streptomyces peucetius* [33]. The *drrA*, *drrB*, and *drrC* efflux pump are parts of the ABC transporter complex involved in doxorubicin resistance [34]. In this study, *drrC* (*Rv2938*) had the highest expression level compared to other genes in the 16 MDR/pre-XDR/XDR isolates, as it was overexpressed in 4 (25%) and 5 (31%) isolates under RIF and INH stress, respectively. On the other hand, the VP-treated MDR/pre-XDR/XDR isolates could down-regulate the *drrC* efflux gene more remarkably than most other genes. In this study, we revealed that only 1 out of the 9 mono-resistant isolates overexpressed *drrC*; whereas, this efflux pump was down-regulated by 3/9 VP-treated mono-resistant isolates. Additionally, in the mono-resistant isolates, *Rv2938* had the highest down-regulation compared to other genes. Gupta et al. demonstrated that *Rv2938* played a potent role in drug resistance, especially toward EMB and STR [35]. The PII36 isolate showed the upregulation of the efflux gene *drrC* by INH and RIF and down-regulation in the presence of RIF + VP. The results of our study suggest that *drrC* plays an important role in the INH/RIF resistance in *M. tuberculosis*.

Choudhuri et al. showed that *drrAB* was expressed in *Mycobacterium smegmatis,* which can contribute to the resistance to EMB, STR, norfloxacin, erythromycin, tetracycline, and chloramphenicol [36]. Our study revealed that RIF and INH induced a four-fold increase in *drrA* (*Rv2936*) expression in 3 out of 16 MDR/pre-XDR/XDR isolates. However, we found that *drrA* was down-regulated in 2 and 3 MDR/pre-XDR/XDR isolates with VP + RIF and VP + INH treatment, respectively. Additionally, our study showed that *drrA* could be up-regulated and down-regulated in PII27 (by INH and RIF) and PII36 (by VP) isolates, respectively. In the presence of VP, the MIC of INH (16 μg/ml) reduced by 64 folds (0.25 μg/ml) in isolate PII27**,** which showed that the efflux activity could be inhibited by VP. In contrast, our study suggested that *drrA* failed to be up-regulated or down-regulated with VP treatment in mono-drug resistant isolates, demonstrating that *drrA* may be one of the drug resistance factors in MDR/pre-XDR/XDR isolates, with no significant contribution to resistance in mono-resistant isolates.

Pang et al. showed that *drrA* might be involved in one of the RIF-related efflux pumps in mono-resistant isolates, which was not consistent with our results [37]. In a similar study, Li et al. reported that *drrB* was up-regulated in 4 and 2 out of 9 MDR isolates in response to INH and RIF, respectively [30]. Our results demonstrated that *drrB* (*Rv2937*) failed to be up-regulated in any of the MDR/pre-XDR/XDR isolates by RIF and INH, while *drrB* was down-regulated in one and none of these MDR/pre-XDR/XDR isolates in response to INH + VP and RIF + VP stress, respectively. In spite of the down-regulation of *drrB* after VP exposure in one of the nine isolates, among the mono-drug resistant isolates, none was significantly up-regulated with RIF, INH, and EMB treatment.

RND transporter as a part of the efflux pump contributes to the *M. tuberculosis* drug resistance. Recently, among the RND transporters, *MmpL* has emerged as an essential key in the elaboration of the cell envelope of mycobacteria. Moreover, a few *MmpL* proteins, such as *MmpL5*, have been demonstrated to participate in the active efflux of antitubercular drugs [38]. *mmpL13a* (*Rv1145*) and *mmpL13b* (*Rv1146*) have been shown to be the variants of an individual gene in the H37Rv strain of *M. tuberculosis* although 14 *mmpL* genes have been discovered in the *M. tuberculosis* genome [39]. Our study demonstrated that among 16 MDR/pre-XDR/XDR isolates, *mmpL13a* and *mmpL13b* were individually overexpressed under INH stress in two strains (PII-28 and PII-31) while they were co-expressed in the PII-33 isolate.

*mmpL13a* and *mmpL13b* were also up-regulated in two and one MDR/pre-XDR/XDR isolates under RIF stress, respectively. This result was not consistent with the findings of a previous report [30]. However, *mmpL13a* and *mmpL13b* were independently down-regulated in two and none of these MDR/pre-XDR/XDR isolates in response to RIF + VP and INH + VP stress, respectively. On the other hand, *mmpL13a* and *mmpL13b* were not up- or down-regulated in any of the nine mono-resistant isolates after RIF, INH, EMB, and VP exposure. This result suggests, for the first time, that *mmpL13a* and *mmpL13b* have higher expression rates in MDR/pre-XDR/XDR isolates compared to mono-drug resistant isolates under RIF and INH stress.

Our study showed that the high-level expression of two specific genes in the MFS, i.e., *Rv1250* and *Rv876*, occurred in two and one of the MDR/pre-XDR/XDR isolates under RIF and INH stress, respectively. On the other hand, efflux pumps *Rv1250* and *Rv876* were down-regulated under VP + RIF stress in one isolate; however, in this study, we revealed that *Rv1250* and *Rv876* were down-regulated by VP + INH treatment in two and none of the MDR/pre-XDR/XDR isolates, respectively.

Contrary to our findings, Narang et al. reported that *Rv1250* and *Rv876* were not overexpressed in any of the MDR isolates due to INH stress [9]. In this study, we revealed that among mono-resistant isolates, only were *Rv1250* and *Rv876* co-overexpressed in the PII-7 isolate in response to RIF. Isolate PII-7 also showed a significant reduction in the expression of *Rv1250* and *Rv876* in the presence of VP, suggesting the role of *Rv1250* and *Rv876* in the RIF resistance of the PII-7 isolate. *EfpA* (*Rv2846c*), another efflux transporter of the MFS family, was overexpressed in two MDR/pre-XDR/XDR isolates (PII-20 and PII-33) in response to RIF or INH stress and in one mono-resistant isolate (PII-18) in response to EMB. The PII-20 isolate showed down-regulation of the efflux genes *Rv2846c*, *Rv2459*, *Rv849*, and *Rv1819,* as well as a drastic eight-fold reduction in the MIC of INH in the presence of VP, confirming the role of efflux pumps in the INH resistance in this isolate. However, Machado et al. found that *EfpA* was upregulated independently of the exposure drugs, which is in concordance with our findings [40].

The efflux pump protein *Rv933* (*pstB*) was also categorized into the ABC transporter family [37]. Our results demonstrated that *Rv933* was overexpressed in three of these MDR/pre-XDR/XDR isolates under RIF and INH stress. Besides, *Rv933* was down-regulated with RIF + VP and INH + VP treatment respectively in two and three of these MDR/pre-XDR/XDR isolates. Although *Rv933* failed to be up-regulated in any of the mono-resistant isolates with RIF, INH, and EMB treatment, *Rv933* was down-regulated in two out of nine mono/poly drug-resistant isolates. In another study conducted by Oh et al., it was reported that the enhanced expression of *pstB* in clinical drug-resistant tuberculosis isolates may contribute to drug resistance in *M. tuberculosis* [41]. However, in this study, we showed that the expression of *Rv1250*, *Rv3239*, *Rv3065*, and *Rv1672* in the presence of RIF and *Rv1410*, *Rv3239*, and *Rv2333* in the presence of INH significantly increased in two MDR/PRE-XDR/XDR isolates. Interestingly, the isolate PII-25 overexpressed the *Rv2333* (*stp*) gene due to INH treatment and down-regulated it in the presence of INH + VP, indicating that this gene is involved in the efflux of INH. Regarding the isolate PII-27 with high initial resistance to RIF (MIC 128 μg/ml), we observed that the RIF MIC reduced (MIC 0.25 μg/ml) in the presence of VP, implying the overexpression of efflux gene*s Rv933*, *Rv1250*, *Rv*2936, *Rv*1410*,* and *Rv*2938 in response to RIF stress. On the contrary, none of these genes was down-regulated by RIF + VP. We speculate that other efflux genes are involved in the development of resistance in this strain. Surprisingly, the isolate PII-18, with resistance to EMB (MIC 3.125 μg/mL) and no mutations at *embA* or *embB*, showed the overexpression of three efflux genes (i.e., *Rv1250*, *Rv2846*, and *Rv2938*) in response to EMB stress. On the other hand, the isolate PII-18 showed a significant reduction in the expression of *Rv1250* and *Rv2938* in the presence of VP, suggesting the role of *Rv1250* and *Rv2938* in EMB resistance in the PII-18 isolate.

The Mmr efflux transporter (*Rv3065*) is the only efflux pump from the SMR family presented in the *M. tuberculosis* genome [42]. We found that this efflux pump was overexpressed in two MDR/pre-XDR/XDR isolates (i.e., PII-28 and PII-31) in response to RIF or INH. The MIC of RIF in the PII-28 isolate reduced by 16 folds in the presence of VP; this efflux pump was among the genes down-regulated under VP treatment.

## Conclusion

Our results clearly show that efflux systems play a role, besides spontaneous mutations, in the development of INH/RIF resistance. Moreover, there were some associations between *Rv2938, Rv2936, Rv1145, Rv1146,* and *Rv933* genes and INH/RIF drugs in the current study. In addition, a similar association was noted between *Rv1250* and *Rv2938* genes and EMB. A direct association was also found between *Rv1250* and *Rv876* genes and RIF resistance, as well as between *Rv2333(stp), Rv2846c, Rv* 2459, *Rv* 849, and *Rv* 1819 genes and INH resistance. Finally, although VP was effective in reducing the expression of some efflux pumps, it was not very successful at the phenotypic level. Determining Time-Kill Curves and in vivo studies are required to confirm our results. More importantly, in spite of the fact that VP is approved by the FDA as EPI, its application as anti-TB drugs still needs further investigation.

## Additional file

Additional file 1: **Table S1**. Differential expression of efflux pump genes under RIF, INH, VP+RIF and VP + INH stress in MDR *M tuberculosis* isolates. **Table S2.** Differential expression of efflux pump genes under RIF, INH, EMB, VP + RIF, VP + INH, and VP + EMB stress in mono-resistant *M tuberculosis* isolates. (XLS 84 kb)

## Abbreviations

ABC: ATP-binding cassette
ADC: Albumin-dextrose-catalase
CAP: Capreomycin
EMB: Ethambutol
EPI: Efflux pump inhibitor
INH: Isoniazid
KAN: Kanamycin
LMM: Linear mixed model
MATE: Multidrug and toxic compound extrusion
MDR: Multi-drug resistant
MIC: Minimum inhibitory concentration
OFX: Ofloxacin
PACE: Proteobacterial antimicrobial compound efflux
RIF: Rifampicin
RND: Resistance–nodulation–division
SMR: Small multidrug resistance
STR: Streptomycin
VP: Verapamil
XDR: Extensively drug-resistant
